# Influence of the Physicochemical and Microstructural Properties on the Insecticidal Efficacy of Diatomaceous Earth on the Poultry Pest *Alphitobius diaperinus*

**DOI:** 10.1021/acsomega.5c06320

**Published:** 2025-10-28

**Authors:** Rayssa Barbary Pedroza Moura, Laís Carneiro Naziasene Lima, Ana Lúcia Coutinho Teixeira, Caio Marcio de Oliveira Monteiro, Fabio Furlan Ferreira, Juliana Pereira da Silva, Ana Luiza Lima, Marcilio Cunha Filho, Stephânia Fleury Taveira, Ricardo Neves Marreto

**Affiliations:** † Laboratory of Nanosystems and Drug Delivery Devices (NanoSYS), School of Pharmacy, Universidade Federal de Goiás (UFG), Goiânia, GO 74605-170, Brazil; ‡ Laboratory of Biology, Ecology and Tick Control, Veterinary and Zootechnics School, Universidade Federal de Goiás (UFG), Goiânia, GO 74690-900, Brazil; § Centro de Ciências Naturais E Humanas (CCNH), 74362 Universidade Federal Do ABC (UFABC), Santo André, SP 09210-580, Brazil; ∥ Laboratory of Food, Drug, and Cosmetics (LTMAC), School of Health Sciences, 28127 University of Brasilia, Brasilia, DF 70910-900, Brazil

## Abstract

Different diatomaceous earth (DE) samples were studied to evaluate the influence of physicochemical and microstructural characteristics on insecticidal activity against *Alphitobius diaperinus*. The insecticidal efficacy study showed that DE4 was more effective for larvae and adults (98% and 88% mortality, respectively), contrasting with DE1, which presented similar results to the control (49% and 6% mortality for larvae and adults, respectively). DE2 and DE3, in turn, exhibited promising insecticidal results. X-ray diffraction analyses showed a predominantly crystalline profile for DE1, while an amorphous profile was found for DE4. Laser diffraction analysis and scanning electron microscopy showed that DE1 had a larger average particle size (∼45 μm) than DE2, DE3, and DE4 (∼28, 26, and 25 μm, respectively). In addition, DE1 showed poor powder adherence to the adult insects and reduced adsorption/absorption capacity compared with the other samples (*p* < 0.05). The calcined treatment promoted significant microstructural changes in DE samples. An increase in the crystallinity of the samples is perceived, accompanied by an increase in particle size and a reduction in their adsorption/absorption capacity. These changes significantly impact the insecticidal activity of DE samples against adult insects, resulting in mortality rates of around ∼10–20%. In summary, the results showed that increasing the degree of crystallinity of DE samples negatively affects their insecticidal activity against *A. diaperinus* and seems to be the DE characteristic that most influences insecticidal activity.

## Graphical abstract

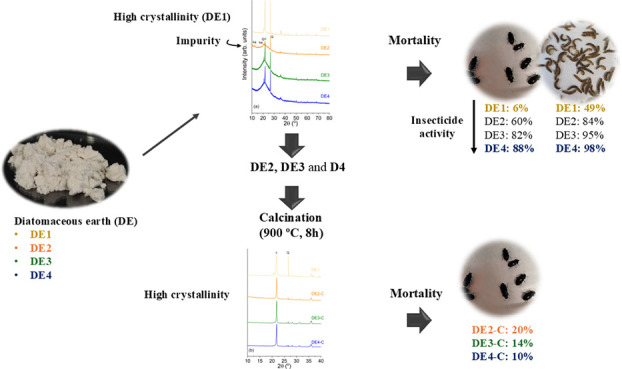

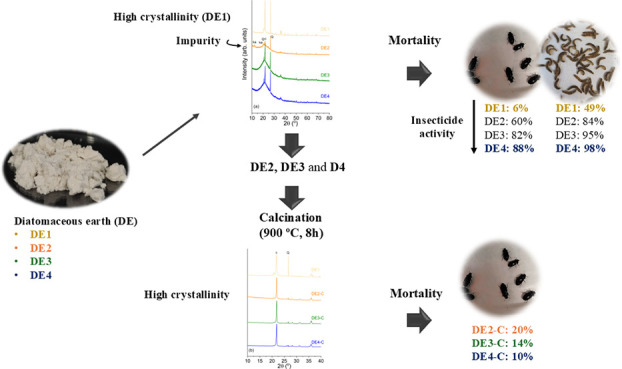

## 1 Introduction

Diatomaceous earth (DE), also known as diatomite and Kieselguhr,^1^ is a type of sedimentary rock that comes from the fossilization of diatom algae, sedimented together with inorganic materials over thousands of years in various aquatic environments.^2,3^ DE is composed mainly of amorphous silicon dioxide (SiO_2_) and, in a minority, of clay, aluminum-based substances, phosphorus, iron, and phosphate. In addition, it contains fractions of organic matter in varying quantities depending on the DE source. DE is generally a porous material with a high capacity to absorb oils, grease, and water, with a size distribution extending from 2 to hundreds of micrometers. It is chemically inert, presenting a large specific surface area, reaching 200 m^2^/g. These properties are related to several industrial applications of DE, including a filtration agent, a mild abrasive, animal feed additive, pesticide carrier, and insecticidal activity.^1,3–8^

DE as an insecticide is well-established, and commercial DE formulations are available to control insects that feed on stored grain. It can be applied to the entire grain mass or used in structural treatments at grain storage sites. Commercial formulations include Insecto, Fossil-Shield, SilicoSec, Perma Guard, Protect-It, Silicon Protect, Diafil 610, among others.^9–12^ Several factors contribute to making DE an insecticide of interest, including low environmental and mammalian toxicity, low potential for the development of insecticidal resistance, low cost, and wide availability.^13–16^ The mechanisms of insecticidal activity encompass abrasion of the insect’s cuticle, obstruction of cuticular pores, and sensilla. In addition, the absorption of cuticular lipid molecules may trigger the death of the insect by desiccation.^2,16,17^ Despite having proven insecticidal activity, many DE samples have shown variable efficacy in different studies, which has been attributed to both the insect’s characteristics and the properties of DE. Particularly, it has been reported that variable efficacy may be related to particle size, particle active surface, pore diameter, moisture content, SiO_2_ content, and compacted density.^2,13–15,18–21^

Commercial DE is a dry and ground material. It is often calcined or flux-calcined depending on the intended application. Calcination is necessary to purify the material, increasing the adsorption/absorption capacity by eliminating impurities that block the porous network.^22^ The calcination temperature applied will depend on the amount and type of impurities to be removed. However, calcination at high temperatures and for an extended time can modify the microstructural and physicochemical characteristics of DE. The most significant changes involve reducing the surface area, changing the shape and geometry of the diatom structure, losing adsorption/absorption functional groups, and changing its compounds from the amorphous phase into a crystalline one.^22–27^

Different studies have investigated the insecticidal activity of DE; however, some controversial results and gaps need to be elucidated, especially regarding the relationship between the material’s physicochemical and microstructural properties and its insecticidal activity. For example, the relationship between DE crystallinity and insecticidal activity is still unknown. A more in-depth understanding of this relationship would make optimizing DE’s insecticidal activity possible. In addition, studies investigating the activity of DE against important pests such as *Alphitobius diaperinus* (Panzer) are scarce. In fact, *A. diaperinus* is one of the most relevant pests to poultry farming worldwide, and its primary control method is based on chemical insecticides.^28^ However, reports of resistance to chemical insecticides have motivated the search for new insect control strategies.^29–31^ As a result, some studies have investigated DE as a physical method for controlling *A. diaperinus*.^32–38^ As it has a physical mechanism of action, insect resistance to DE is unlikely.^13^ In addition, DE is a highly abundant and low-cost inorganic material globally, making it feasible to use as an insecticide to control *A. diaperinus*. In this study, an in-depth characterization of DE samples from different sources was conducted to clarify the relationship between DE’s microstructural properties and its insecticidal activity for *A. diaperinus*.

## 2 Materials and Methods

### 2.1 Reagents and Insects

Methylene blue (MB) was purchased from Neon Comercial Reagentes Analíticos Ltd. (São Paulo, Brazil). Mineral oil was purchased from Tapcamp Soluções Industriais Ltd. (São Paulo, Brazil).

The larval and adult specimens of *A. diaperinus* were collected at the Aviary School of the Veterinary School (EVZ) of the Federal University of Goiás (UFG). After collection, the specimens were taken to the Tick Biology, Ecology, and Control Laboratory (LABEC) at the EVZ, where colonies were reared. Larvae and adults were separated and kept in plastic boxes (55 cm × 35 cm × 27 cm) at room temperature and humidity until the experiments were carried out. In addition, corn grits were selected for feeding the insects, and pieces of apple were added to the colonies of larvae and adults at least twice a week to serve as a source of moisture.

### 2.2 DE Samples

The DE samples were obtained from different suppliers: Isofar Indústria e Comércio de Produtos Químicos Ltd. (Rio de Janeiro, Brazil), DE1; Biomarkan Mineração Industrial Ltd. (Aquiraz, Brazil), DE2; Bequisa Indústria Química do Brasil Ltd. (São Vicente, Brazil), DE3; Ciemil Comércio Indústria e Exportação Ltd. (Vitória da Conquista, Brazil), DE4 (Table 1). According to suppliers’ information, DE2 is natural (nonprocessed by calcination), DE4 is calcined (900 °C), and DE1 and DE3 have an unknown processing history.

**Table 1:** Properties of the Diatomaceous Earth Samples

Samples	Code	Mean size ± SD (μm)	Supplier
Nonprocessed samples	DE1	45.83 ± 26.80	Isofar Ltd.
	DE2	28.05 ± 26.52	Biomarkan Ltd.
	DE3	26.65 ± 18.20	Bequisa Ltd.
	DE4	25.23 ± 17.34	Ciemil Ltd.
Calcined samples	DE2-C	209.1 ± 246.9	Biomarkan Ltd.
	DE3-C	59.25 ± 75.61	Bequisa Ltd.
	DE4-C	39.19 ± 52.15	Ciemil Ltd.

In the present study, selected samples (DE2, DE3, and DE4) were submitted to a calcination process at 900 °C for 8 h in a muffle furnace. After calcination, the samples were ground with a mortar and pestle. The mean size of the DE samples was determined by laser diffraction using a particle size analyzer LS 13320 XR (Beckman Coulter, Brea, CA, USA). The samples were dispersed in mineral oil for 30–60 s before analysis (*n* = 3).

### 2.3 Insecticidal Activity of Diatomaceous Earth (DE) on Larvae and Adults of *Alphitobius diaperinus*

The insecticidal activity of the nonprocessed DE was evaluated on larvae and adults of *A. diaperinus*. The experiment was carried out in Petri dishes (6 cm in diameter) containing a mixture of milled corn and DE (100, 200, and 400 mg of DE/6 g of corn bran). For each DE concentration, ten Petri dishes containing five insects each were used (*n* = 50). The control sample contained only the substrate without DE.

Corn bran mixtures with DE samples were prepared by adding the proper DE mass to Petri dishes containing the substrate, followed by circular movements. Next, the insects were placed on Petri dishes. The samples were kept in a BOD chamber EL 111/3 (Eletrolab, São Paulo, Brazil) (26 ± 1 °C and 64 ± 26% RH) for 10 days for adults and 8 days for larvae. The death count (absence of movements) was carried out every 2 days with the aid of a stereomicroscope. Calcined samples (Table 1) were also evaluated for adult insecticidal activity using the same protocol (400 mg of DE/6 g of corn bran).

### 2.4 Fourier Transform Infrared Spectroscopy (FTIR)

FTIR analysis of nonprocessed and calcined DE samples was carried out in the mid-infrared region (4000 to 400 cm^–1^) using the diffuse reflectance technique. A Spectrum 400 spectrophotometer (PerkinElmer, Waltham, MA, USA) was used for this purpose.

### 2.5 Scanning Electron Microscopy (SEM)

SEM analysis of nonprocessed and calcined DE samples was carried out using a scanning electron microscope JSM 6610 (Jeol, Tokyo, Japan). Samples were coated with gold using the Desk V gold film deposition system (Denton Vacuum LLC, New Jersey, USA) and analyzed under different magnifications.

### 2.6 X-ray Diffraction (XRD) Analysis

XRD analysis of nonprocessed and calcined DE samples (Table 1) was carried out in a STADI-P diffractometer (Stoe, Darmstadt, Germany). Monochromatic radiation wasgenerated in a copper anode tube coupled to a Johann-type monochromator (Ge(111) curved crystal) for *K*α_1_, operating at 40 kV and 40 mA, in the 2θ range from 10.000° to 93.335°, with a step of 0.015° and a counting time of 50 s at each 1.05°.

### 2.7 Thermogravimetric Analysis (TGA)

The TG analysis was conducted in an STA 449 F3 Nevio simultaneous thermal analyzer (Netzsch, Selb, Germany). About 10 mg of nonprocessed samples (Table 1) were placed in an alumina crucible and analyzed under carefully controlled conditions. The temperature was varied from 30 to 900 °C at a heating rate of 10 °C/min under a controlled flow of nitrogen (40 mL/min) and oxygen (10 mL/min).

### 2.8 Powder Distribution of Diatomaceous Earth (DE) on the *A. diaperinus*

DE samples (0.2 g) were manually mixed with corn bran (3 g), as described in Section 2.3. Next, five insects were placed in the plates and kept for 10 days at room temperature. The control sample did not contain DE. At the end of the experiment, the dead insects were analyzed under a stereomicroscope S9i (Leica Microsystems, Wetzlar, Germany) and photographed.

### 2.9 Adsorption and Absorption Analysis of Methylene Blue (MB) in Diatomaceous Earth (DE)

Nonprocessed and calcined DE samples had their dye adsorption capability investigated by using MB as the adsorbate. An MB analytical curve (0.75–6.0 μg/mL) was constructed in a Genesys 50 UV–vis spectrophotometer (Thermo Fisher Scientific, Waltham, MA, USA) at 664 nm. Next, 8 mL of an MB solution (0.1 mg/mL) was added to a beaker containing 100 mg of each DE. The mixture was magnetically stirred for 120 min at 800 rpm (RT 15, IKA, Staufen, Germany). At predetermined times (30 and 120 min), 1 mL of the mixture was withdrawn and centrifuged at 3000 rpm for 10 min (Solab, SL-702, São Paulo, Brazil), and the supernatant was quantified. The MB adsorption/absorption capability was calculated (*n* = 3) according to eq 1

1 $$C_{a}\left(mg/g\right)=\frac{\left(C_{0}-C_{t}\right)\times V\left(L\right)}{m\left(g\right)}$$

where *C*_a_ is the concentration of MB adsorbed/absorbed in the DE (mg/g), *C*_0_ is the initial concentration of MB (mg/L), *C*_*t*_ is the concentration of MB quantified in the supernatant at each time evaluated (30 and 120 min), *V* is the volume of the MB solution, and *m* is the mass of DE used.

### 2.10 Nitrogen Adsorption and Desorption Analysis

Nitrogen adsorption and desorption analyses were conducted to characterize the nonprocessed DE samples in terms of surface area, pore diameter, and pore volume. The surface area was determined by the Brunauer–Emmett–Teller (BET) model, and the diameter and pore volume was determined by the Barrett–Joyner–Halenda (BJH) method, using the ASAP 2020 adsorption analyzer (Micromeritics, Norcross, GA, USA). The samples were dried for 6 h and analyzed by using nitrogen as the adsorbate. The analyses were performed at 30 °C.

### 2.11 Analysis of Water Content of the Diatomaceous Earth (DE) Samples

The nonprocessed DE samples (1 g) were placed on an infrared balance LJ16 (Mettler Toledo, Columbus, OH, USA), and the material was heated to 110 °C until a constant weight (*n* = 3).

### 2.12 Statistical Analysis

The statistical analysis of the mortality data gathered from insecticidal activity tests was carried out using R software, version 4.3.0. The Kruskal–Wallis test for multiple comparisons was used. Other statistical analyses were carried out using ANOVA or Student’s *t* test, considering *p* < 0.05 as the minimum significance level.

## 3 Results

### 3.1 Insecticidal Activity of DE

Mortality caused by the DE1 sample was similar to the control one (*p* > 0.05). On the other hand, the other DE samples produced considerably higher mortality results for both larvae and adult insects than the control group (*p* < 0.05). Moreover, mortality was concentration-dependent. In particular, higher insecticidal activity was obtained with DE4 compared to DE2 (*p* < 0.05) at the highest concentration. Finally, the DE samples at the highest concentration that were calcined (DE2-C, DE3-C, and DE4-C) showed a significant reduction in their insecticidal activity in adults (*p* < 0.05). Calcined samples were not tested against larvae since we aimed to elucidate whether thermal treatment led to a reduction in biological activity. Assays performed with adults had already supplied sufficient evidence.

The mortality of the adults from DE2, DE3, and DE4 treatments ranged from 20 to 88%, while from DE1 treatment, it was lower than 10%, with no statistical difference compared to the control group (*p* *>* 0.05). The percent mortality of adults was lower than that observed for larvae, likely due to the differences in the susceptibilities of these different *A. diaperinus* stages. Notably, a concentration-dependent activity was observed not only for DE3 and DE4 but also for DE2, underscoring the role of dosage in the effectiveness of the treatments. Generally, the percent mortality significantly increased when the DE concentration increased from 100 to 400 mg/plate. Other studies have also observed this behavior using DE as an insecticidal agent.^13,39^

### 3.2 FTIR of Calcined and Nonprocessed DE Samples

Typical vibration bands in DE samples could be identified in the spectra presented in Figure 1. At 480 cm^–1^, the band ascribed to Si–O stretching^26^ can be observed in all samples. At 610 cm^–1^, the characteristic vibration of the cristobalite tetrahedron^40^ was observed only for the DE1 sample, in agreement with the crystalline nature of this material (Section 3.4). Vibration of the Si–O–H bond can be seen at 700 and 800 cm^–1^,^24,26^ whereas Si–O stretching of the silanol group was observed at 910 cm^–1^.^24^ The bands at approximately 1100 and 1630 cm^–1^ were attributed to the stretching of the siloxane group (Si–O–Si).^8,24^ The band at 3400 cm^–1^ represents the O–H bending of the water molecules weakly adsorbed on the DE surface.^7,8,24^ Lastly, the bands at approximately 3610 and 3700 cm^–1^ were associated with the free silanol group (SiO-H).^24^

**Figure 1:**
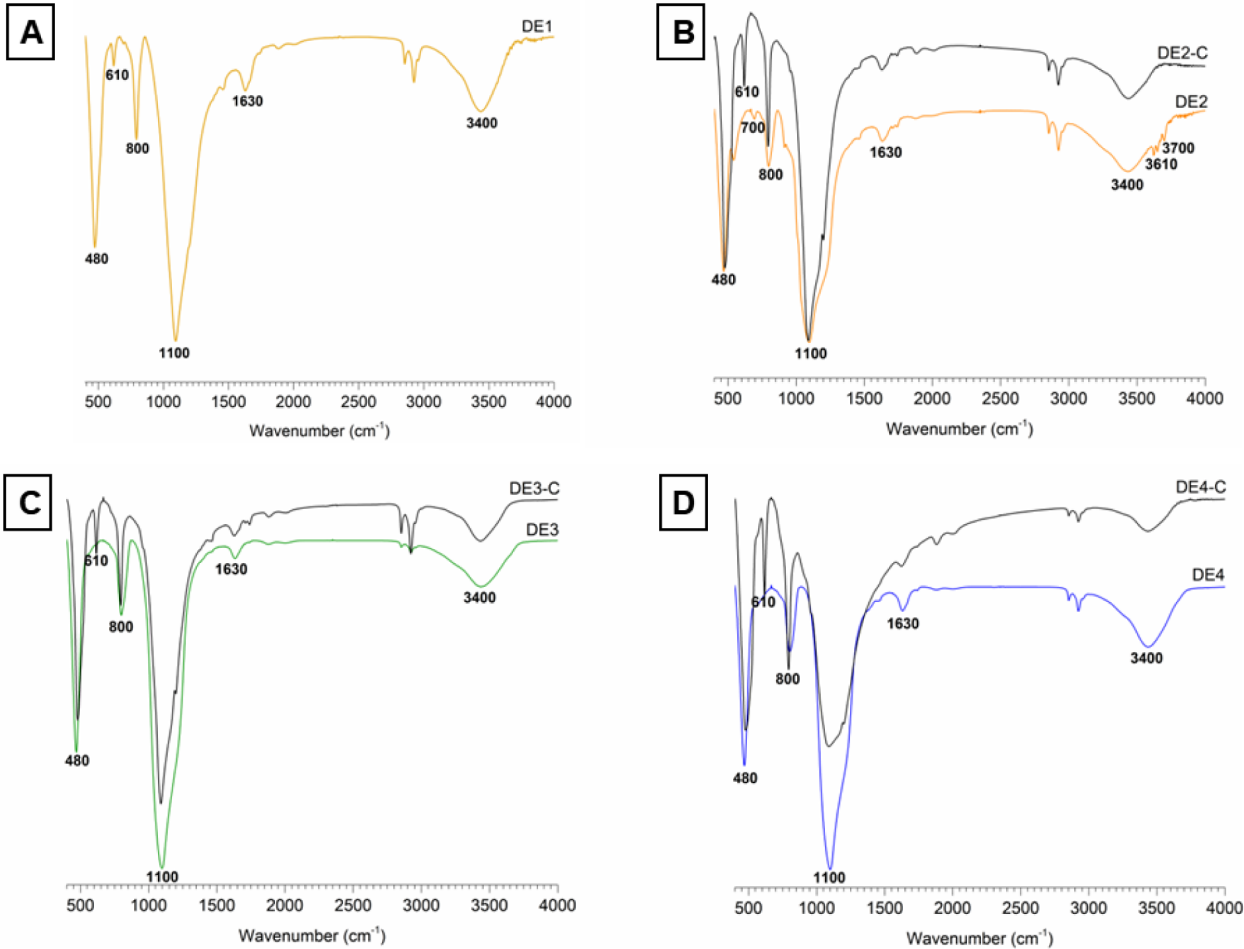
Fourier transform infrared spectroscopy of nonprocessed and calcined samples. (a) DE1 spectrum; (b) DE2 and DE2-C spectra; (c) DE3 and DE3-C spectra; (d) DE4 and DE4-C spectra.

The spectra of the calcined samples (DE2-C, DE3-C, and DE4-C) showed spectral changes denoted by the band’s appearance at 610 cm^–1^, indicating the presence of cristobalite. Additionally, the disappearance of the bands at 700, 3610, and 3700 cm^–1^ in DE2-C suggested the loss of the silanol groups.

### 3.3 SEM and Size Analysis

SEM micrographs of the nonprocessed and calcined DE samples can be seen in Figure 2. It can be seen that DE2, DE3, and DE4 have particle sizes that are lower than those of DE1, which agrees with the laser diffraction data presented in Section 2.2. The micrograph of the calcined sample DE2-C showed both an increase in particle size and a more irregular size distribution compared with DE2, which is also in agreement with laser diffraction data and suggests the occurrence of particle agglomeration during the thermal processing. On the other hand, the differences between DE3/DE3-C and DE4/DE4-C were less pronounced, but a slight increase in particle size could still be seen. Furthermore, all the samples have irregularly shaped particles, but DE2-C, DE3-C, and DE4-C samples have a rougher surface, similar to the surface of the DE1 sample, while DE2, DE3, and DE4 samples have a smoother surface. In addition, they have many chip-shaped particles, probably fragments of diatom algae frustules.

**Figure 2:**
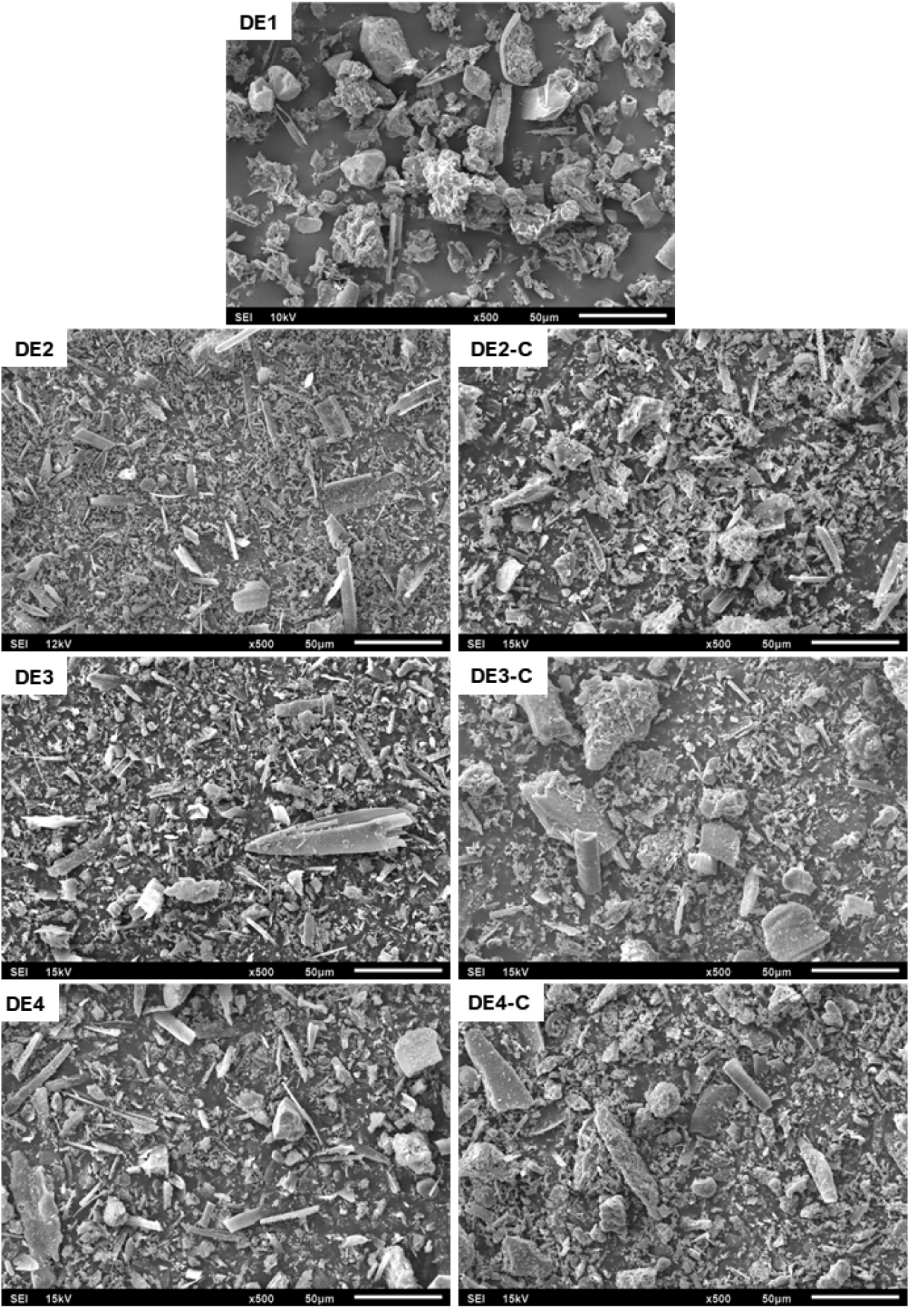
Micrographs of non-processed (DE1, DE2, DE3, and DE4) and calcined samples (DE2-C, DE3-C, and DE4-C) at 500× magnification.

### 3.4 XRD Analysis of Nonprocessed and Calcined DE Samples

The DE samples were analyzed by using XRD (Figure 3). Phase identification was conducted using a search-match procedure in the Qualx2 software,^41^ employing a compiled version of the Crystallography Open Database (COD).^42^ The identified crystalline phases were matched with the following COD entries: cristobalite (9008227), quartz (1011172), sodium sulfate (thenardite, 9004092), and kaolinite (1011045). Quantitative phase analysis (QPA)^43,44^ and structural characterization were performed through Rietveld refinements^45^ using the TOPAS-*Academic* v7 software.^46^ Rietveld refinements of all samples are displayed in the Supporting Information. During the Rietveld refinement, the lattice parameters and isotropic atomic displacement parameters were refined for each crystalline phase. The background radiation was modeled using a 5-term Chebyshev polynomial, which was previously determined from the refinement of a silicon standard reference material (NIST) and subsequently held fixed during the analysis of the samples. To account for the amorphous contribution, primarily from the semicrystalline cristobalite, an *hkl* phase was introduced, defined with an orthorhombic unit cell and lattice parameters of *a* = *b* = 0.8 Å, and *c* = 40 Å. This approach is typically employed for quantitative analysis when an internal standard is not used. The degree of crystallinity for each sample was then calculated as the ratio of the integrated area of the crystalline phases to the total scattered area (crystalline + amorphous), according to the expression described in ref 47.

**Figure 3:**
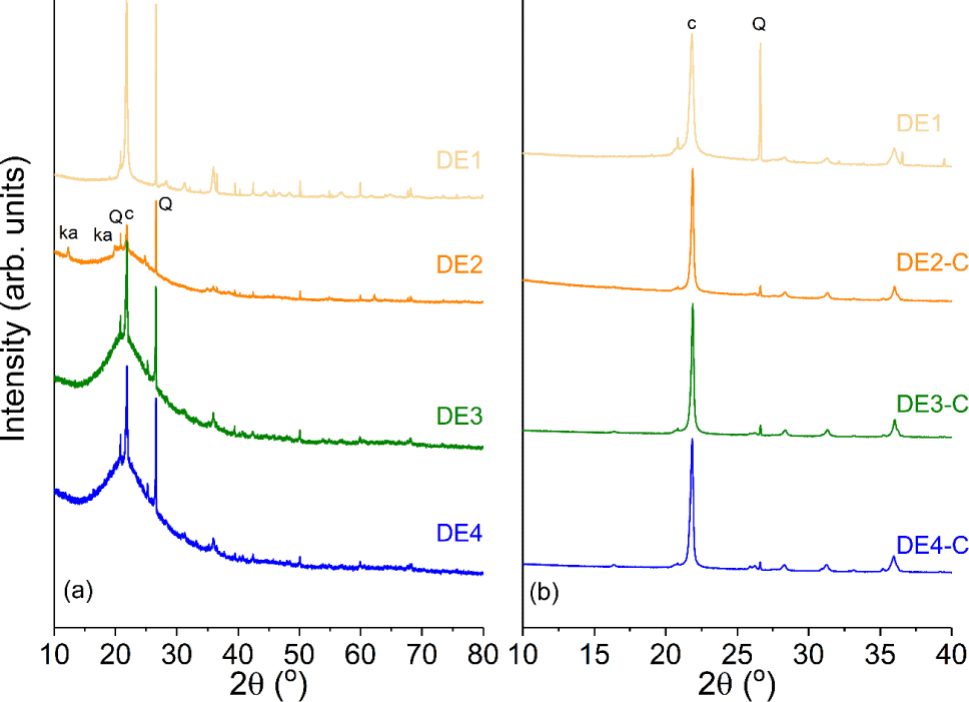
Diffractogram of DE samples: (a) nonprocessed DE samples; (b) calcined samples. Q = quartz; c = cristobalite; and ka = kaolinite.

The mineralogical compositions of the four as-received commercial DE samples revealed significant differences. Sample DE1 is highly crystalline, consisting predominantly of cristobalite (∼68 wt %), quartz (∼21 wt %), and thenardite (Na_2_SO_4_) (∼11 wt %). In contrast, samples DE2, DE3, and DE4 are composed of semicrystalline cristobalite, quartz, and kaolinite, with a substantial amorphous component (Figure 3a). The XRPD pattern of DE2 indicated the presence of kaolinite with notable stacking faults, as evidenced by the characteristic broadening and asymmetry of its diffraction peaks. Samples DE3 and DE4 presented a progressively higher amorphous content when compared to DE2 and a significantly greater amorphous fraction than highly crystalline DE1.

Following calcination at 900 °C, the resulting samples (DE2-C, DE3-C, and DE4-C) underwent a significant phase transformation (Figure 3b). The broad features associated with semicrystalline cristobalite sharpened, indicating a transition to a more crystalline form. Quartz remained as a stable phase. Notably, the diffraction peaks corresponding to kaolinite disappeared, and new peaks corresponding to the formation of mullite (Al_6_Si_2_O_13_) were identified in all three calcined samples.

The results indicated that the nonprocessed samples (DE2, DE3, and DE4) presented a high amount of amorphous contribution, mainly from the cristobalite phase. The degree of crystallinity was ∼5% for all samples (amorphous ∼ 95%). On the other hand, the samples calcined at 900 °C displayed a degree of crystallinity of ∼92% (amorphous ∼ 8%).

### 3.5 TGA of DE Samples

TG curves of the DE samples are presented in Figure 4. The first mass loss stage in TG curves (50–250 °C) is mainly related to the loss of physically bound water. DE2 showed the most pronounced mass loss (6.14%), whereas DE3, DE4, and DE1 showed 2.34, 1.78, and 1.21% mass loss, respectively.

**Figure 4:**
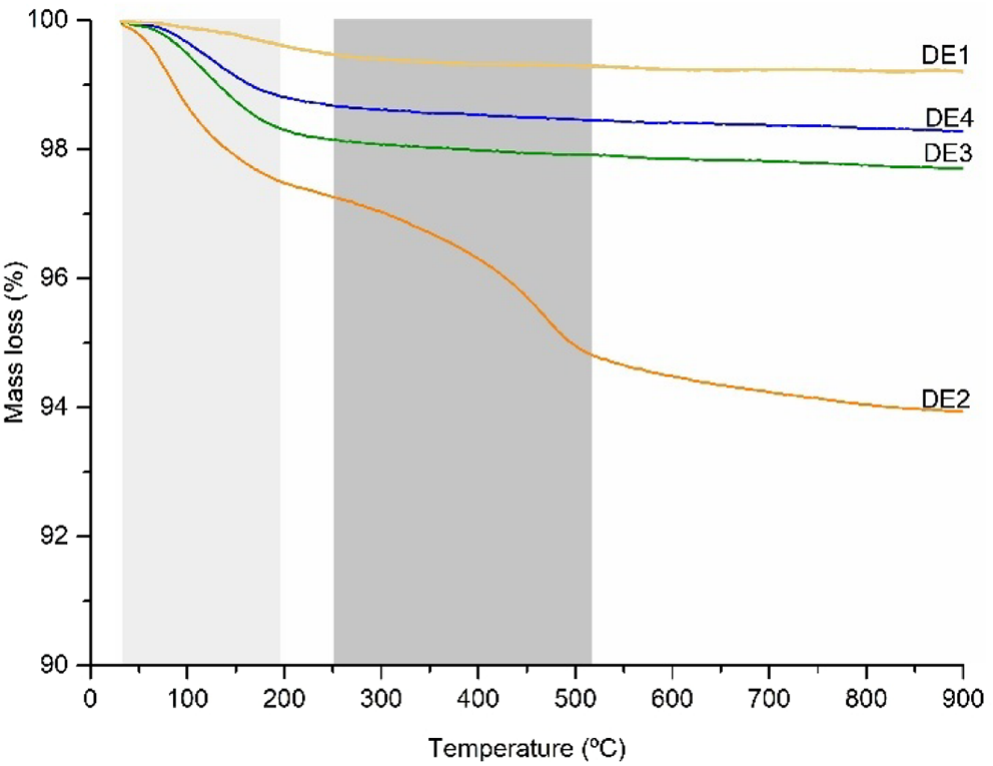
Thermogravimetric curves of non-processed DE samples. First mass loss stage: 50–250 °C (light gray), and second mass loss stage: 300–500 °C (dark gray).

A second mass loss step (300–500 °C) observed in the DE2 curve may be related to the dehydroxylation of kaolinite, a mineral impurity present in some DE samples, and the loss of chemically bound water.^8,48^ This mass step may also be related to organic matter, as reported in ref 48. According to the manufacturer, DE2 has not undergone any calcination process and is characterized as raw DE. DE4 is a calcined sample, which may explain the absence of this sample’s second mass loss stage. Similarly, DE1 and DE3 samples lack the second stage of mass loss, suggesting that those samples also underwent a previous calcination process.

### 3.6 Evaluation of DE Powder Distribution on Adults of *Alphitobius diaperinus*

The adherence and distribution of the control and nonprocessed DE samples (DE1 to DE4, Table 1) on the adult insects were evaluated using a stereomicroscope (Figure 5). The DE2, DE3, and DE4 samples adhered more to the ventral side compared with the DE1 and control samples.

**Figure 5:**
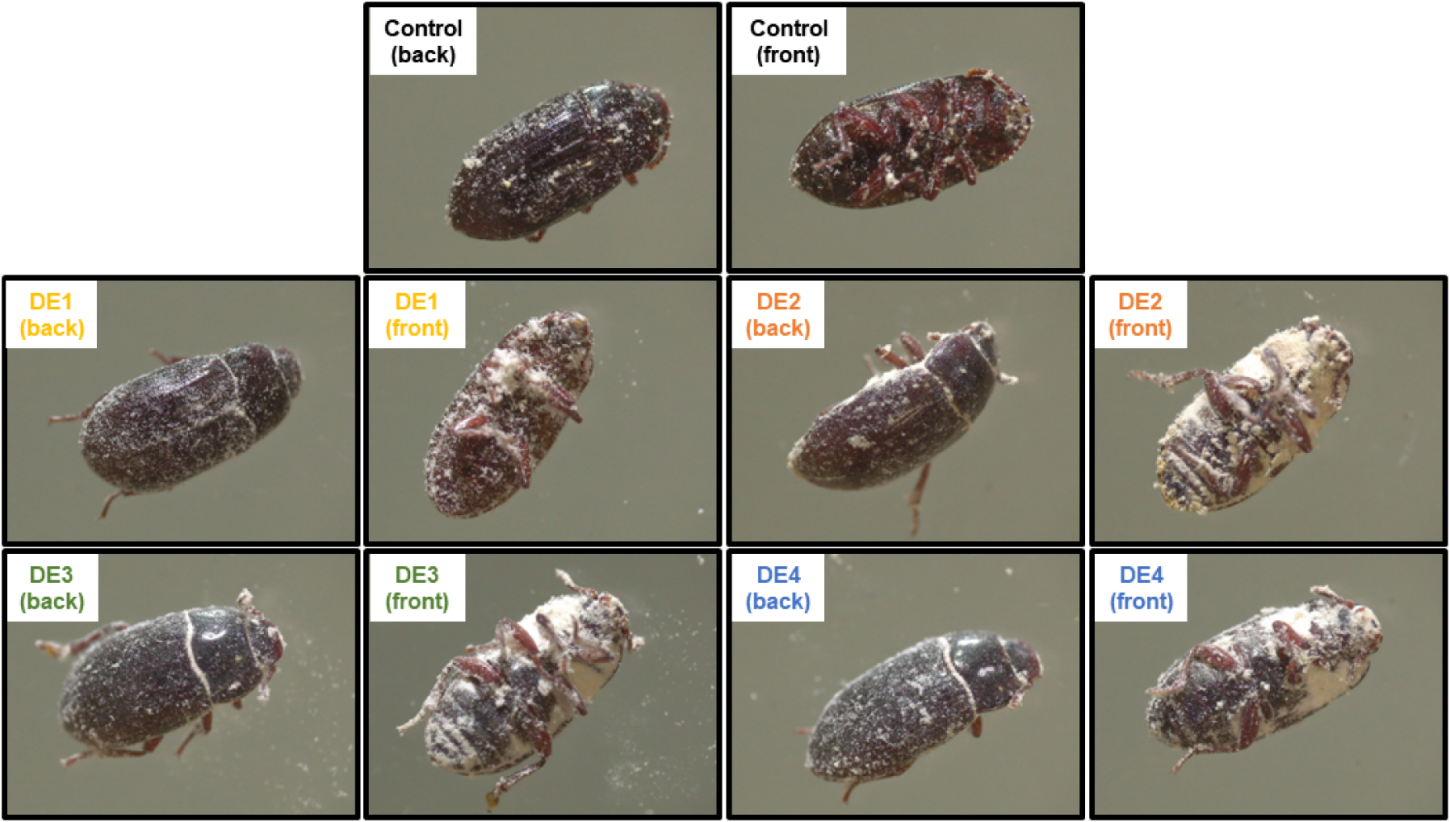
DE powder distribution on *Alphitobius diaperinus*.

### 3.7 Methylene Blue Adsorption/Absorption Analysis

DE samples were also evaluated regarding their ability to adsorb methylene blue dye (MB) (Figure 6). The DE1 sample showed significantly lower dye adsorption at 30 min than the DE2, DE3, and DE4 samples (*p* < 0.05). The same behavior was observed after 120 min incubation (data not shown). There were no statistical differences between DE2, DE3, and DE4 samples (*p* > 0.05).

**Figure 6:**
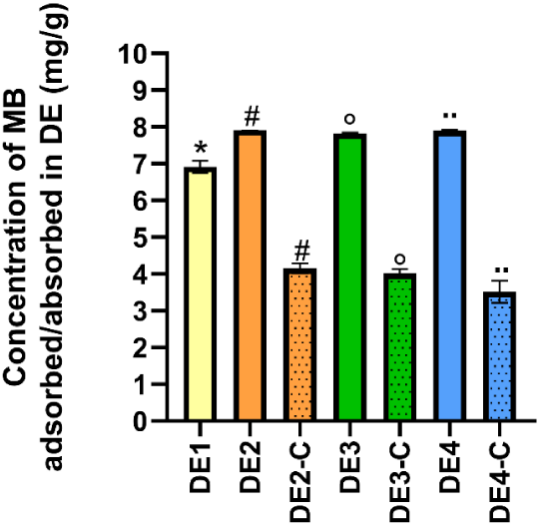
Methylene blue (MB) adsorption from non-processed and calcined DE samples at 30 min. *DE1 is significantly lower than DE2, DE3, and DE4 (*p* < 0.05). Pairs of equal symbols mean that nonprocessed samples and their calcined counterparts were different (*p* < 0.05).

To further understand these relations, we studied the calcined samples’ adsorptive/absorptive capacity (DE2-C, DE3-C, and DE4-C). Figure 6 vividly illustrates that the calcined samples lost their ability to adsorb/absorb MB compared to their nonprocessed samples (*p* < 0.05).

### 3.8 Evaluation of Nitrogen Adsorption/Desorption from Nonprocessed DE Samples

The surface area and porosity-related characteristics (pore volume and diameter) of the nonprocessed DE samples are shown in Table 3.

According to the literature, all DE samples studied here are mesoporous materials with pore diameters between 2 and 50 nm.^49^ In addition, it can be seen that the DE2 sample has the smallest pore volume and diameter. Interestingly, DE1 has pore volume and diameter values similar to those of DE3 and DE4.

## 4 Discussion

Many studies in the literature have shown the insecticidal activity of DE on different species of arthropods, including *A. diaperinus*.^32–34,36–38^ However, relevant variations in the insecticidal efficacy of DE have been observed, mainly attributed to the differences in the physicochemical and microstructural properties of the DE samples.^13,18,19^ The present work studied the insecticidal activity of DE samples obtained from different sources against *A. diaperinus* larvae and adults.

This study showed noticeable insecticidal activity of DE, which was far superior to that of the controls for both larvae and adult stages, except for the DE1 sample. It is important to note that mortality in the control group was similar to that reported in other studies with *A. diaperinus* using similar experimental conditions.^50–52^

It is worth noting the relevant differences in the activity of DE3 and DE4 treatments compared to DE1. The latter showed a percent mortality far below what is expected for an insecticidal material, especially when tested on adult insects. A proper understanding of the relationship between the physicochemical and microstructural properties of the samples and their insecticidal activity is of the utmost importance. Therefore, the samples were processed by calcination (Table 1), a regular purification process, and their insecticidal activity was evaluated. It can be seen that these calcined samples (DE2-C, DE3-C, and DE4-C) had their insecticidal activity drastically reduced compared with the nonprocessed counterparts (*p*< 0.05), practically equaling them to DE1 (Table 2). These findings suggest that calcination can be crucial for insecticidal activity. Therefore, a physicochemical and microstructural characterization of the DE samples was performed to clarify this relation.

**Table 2:** Mortality Percentage of Larvae and Adults of *Alphitobius diaperinus* Exposed to Different Concentrations of Diatomaceous Earth[Table-fn tbl2fn1],[Table-fn tbl2fn2],[Table-fn tbl2fn3],[Table-fn tbl2fn4]

		DE mass in the Petri dish (mg)		
Stages of *A. diaperinus*	Treatments	100	200	400
Larvae	**Control**	22 ± 14%^**A**^	22 ± 14%^**A**^	22 ± 14%^**A**^
	**DE1**	53 ± 21%^**Ba**^	44 ± 23%^**Aa**^	49 ± 23% ^**Aa**^
	**DE2**	78 ± 17%^**Ca**^	89 ± 14%^**Ba**^	84 ± 17%^**Ca**^
	**DE3**	76 ± 15% ^**Ca**^	89 ± 10% ^**Bab**^	95 ± 9%^**BCb**^
	**DE4**	82 ± 14%^**Ca**^	95 ± 13%^**Bb**^	98 ± 6%^**Bb**^
Adult	**Control**	2 ± 6%^**A**^	2 ± 6%^**A**^	2 ± 6%^**A**^
	**DE1**	10 ± 14%^**ABa**^	2 ± 6%^**Aa**^	6 ± 10%^**Aa**^
	**DE2**	20 ± 25%^**ABa**^	30 ± 25%^**Ca**^	60 ± 22%^Cb*^
	**DE3**	38 ± 26%^**Ba**^	74 ± 25%^**Bb**^	82 ± 15%^**BCb#**^
	**DE4**	22 ± 27%^**ABa**^	68 ± 18%^**Bb**^	88 ± 14%^**Bb°**^
	**DE2-C**	-	-	20 ± 27% ^A*^
	DE3-C	-	-	14 ± 27% ^**A#**^
	**DE4-C**	-	-	10 ± 14% ^**A°**^

i The mortality was expressed as the mean ± standard deviation .

ii The symbols *, #, ° represent differences between the non-processed and their respective calcined samples.

iii Different lowercase letters on the same line mean a difference in mortality between the same sample in different concentrations, for larvae and adults.

iv Different capital letters in the same column mean a difference in mortality between samples, for larvae or adults.

Visual assessment of the adherence of DE samples on adult insects showed relevant differences between the samples, which illustrates the differences in insecticidal activity. The DE1 sample, which exhibited negligible insecticidal activity with similar mortality rates to the control (Section 3.1), demonstrated a lower adherence to the insect’s cuticle. DE insecticidal action has been related to lipid extraction from the epicuticular layer, which depends on the powder adsorption on the insect’s cuticular surface.^9,53^ Prasantha et al.^9^ demonstrated this mechanism, reporting DE’s absorption of epicuticular lipids in two insect species. These authors showed that DE could extract from 5 to 63% of the epicuticular fatty acids, which can create intermolecular spaces in the structure, promoting insect desiccation. Insects can die when they lose around 28–35% of their body weight, for instance, caused by lipid and water loss resulting from the adherence of DE on the insects’ surface.^9,53^

The differences in DE’s adherence to the insects’ bodies could be attributed to the size of the DE particles. As evidenced by the SEM micrographs and laser diffraction data, DE2, DE3, and DE4 samples have particle sizes smaller than those of DE1, confirming such a hypothesis.

DE particle size can affect insecticidal activity, as Baliota and Athanassiou^15^ and Vayias et al.^19^ reported. These authors reported that smaller mean particle sizes were more effective against most of the insect species studied. Smaller particles likely adhere to the insect’s cuticle to a greater extent than larger particles, distributing themselves and attaching themselves more easily to the insect; however, it should be taken into consideration that particle size is not the only factor that can affect insecticidal activity.

It is important to note that DE adsorption may have an effect other than promoting desiccation. DE may adhere to the insect’s antennae, as seen for DE2, DE3, and DE4 samples in Figure 5. The antennae of this insect have a sensilla responsible for sensing humidity.^54^ Some authors have already observed that inorganic powders, including DE, can cause deformation and obstruction of sensilla, compromising the insect’s behavior and physiology. Damage to the sensilla, for instance, can lead to reduced food consumption by the insect.^11,54,55^ Accordingly, the DE effects on *A. diaperinus* should be related to desiccation and blockage of hydro receptors, both depending on particle adherence to the insect’s surface. Despite the relevance of particles’ adherence to insects, as demonstrated here, other physicochemical and microstructural factors may have a role in insecticidal activity and were also investigated.

The structural characterization conducted via X-ray powder diffraction of the four commercial DE samples reveals significant compositional heterogeneity, which has direct implications for their thermal behavior and, potentially, their insecticidal efficacy.^56^ Sample DE1 stands out due to its highly crystalline nature and the absence of clay minerals. The presence of thenardite (sodium sulfate) is also unique to this sample, suggesting either a different geological origin or a specific fluxing agent used during its processing.^57^ This high degree of crystallinity could influence its physical properties, such as particle hardness. In contrast, samples DE2, DE3, and DE4 are more representative of natural DE, characterized by a dominant amorphous or semicrystalline silica (cristobalite) phase and the presence of kaolinite as a common clay impurity. However, the supplier of DE4 has informed us that it was calcined at 900 °C. The higher amorphous content observed in DE3 and DE4 might suggest a potentially greater intrinsic insecticidal activity in their as-received state compared to the more crystalline DE1. The stacking faults observed in the kaolinite from sample DE2 indicate a poorly crystalline or disordered structure.^58^

The phase transformations induced by calcination at 900 °C are consistent with the established thermal reactions for aluminosilicate materials. The conversion of the semicrystalline cristobalite to a more ordered, crystalline structure is an expected thermal event. More significantly, the transformation of kaolinite into mullite is a key finding. This conversion typically proceeds via the formation of an amorphous metakaolin intermediate (Al_2_Si_2_O_7_) at ∼550–600 °C, followed by the nucleation of mullite at temperatures around 900–1000 °C. The presence of mullite in samples DE2-C, DE3-C, and DE4-C confirms that the kaolinite impurity was effectively converted.^59^

These transformations have profound implications for the material’s final properties. While calcination increases the overall crystallinity of the silica phase (cristobalite), which might reduce its absorptive capacity,^60^ it also leads to the formation of mullite. Mullite is known for its high hardness and acicular (needle-like) crystal habit.^61^ The introduction of these hard, sharp microcrystals could enhance the abrasive mechanism of insecticidal action, whereby the DE particles physically damage the insect’s cuticle. Therefore, calcination creates a composite material where the insecticidal mechanism might shift from being primarily absorptive to a combination of absorption (from the remaining silica) and abrasion (from the newly formed mullite and sharpened cristobalite). Calcination can be used to engineer a final product with a new set of crystalline phases, such as mullite, whose impact on insecticidal bioassays warrants further investigation.

The time and temperature used in the calcination procedure may define the final properties of DE, consequently affecting its insecticidal properties. The DE1 diffraction pattern is often observed in calcined samples subjected to temperatures exceeding 1100 °C. In this case, the amorphous region is wholly converted into cristobalites.^7,8,26^ Reka et al.^8^ investigated the crystalline conversion of a DE sample for 1 h and showed that DE heating at 1000 °C was not enough to promote the conversion; however, heating the sample at 1100 °C completely transformed the amorphous phase into cristobalite.

In the present study, the calcined samples (DE2-C, DE3-C, and DE4-C) showed a loss of the amorphous structure and the formation of cristobalite (crystalline phase), consequently showing a loss of insecticidal efficacy (Section 3.1). This finding suggests that crystallinity can significantly influence the insecticidal activity of the samples, possibly by hindering intermolecular interactions between DE particles and the insect. The DE4 sample was more effective against *A. diaperinus* than the DE2 sample (*p* < 0.05). As reported by the supplier, the DE4 sample was calcined at 900 °C (for a nonspecified time). Some studies have shown that DE calcination at 900 °C for up to 3 h did not promote cristobalite formation. On the other hand, the authors have observed that calcination up to 900 °C can improve DE’s microstructural properties, removing impurities that block pores^8,25^ and improving its ability to absorb/adsorb substances.^26,62^ These findings may explain some differences observed between DE4 and DE2 samples since DE2 has not undergone a calcination process and has more impurities, evidenced by the presence of the kaolinite (a crystalline clay) diffraction peak in its diffractogram. In sum, impurities and higher crystallinity seem to be related to the lower insecticidal activity in the DE samples.

The DE absorption capacity for the MB dye was evaluated. The results showed a lower adsorption capacity for DE1 compared to those of DE2, DE3, and DE4 samples. As previously described, the DE1 sample showed the lowest insecticidal activity (Section 3.1) and a more crystalline pattern (section 3.4). Higher crystallinity and a larger particle size may affect the adsorption capacity, which, in turn, can explain the lower insecticidal activity of DE1. Indeed, DE2-C, DE3-C, and DE4-C samples also showed a significant decrease in MB absorption compared to their nonprocessed counterparts (DE2, DE3, and DE4).

Additionally, the FTIR analysis of the calcined samples (Figure 1) was carried out to identify any changes in the bands related to silanol or hydroxyl groups. Silanol groups can react with various molecules, including polar organic compounds and a range of functional groups,^23^ being an active site for binding the MB since the nitrogen atoms of MB can form hydrogen bonds with the hydroxyl of the silanol groups, promoting adsorption and/or absorption of the dye.^23,26^ When the DE is heated, silanol groups can dehydrate, reducing MB’s interaction with the DE. Additionally, isolated hydroxyl groups (−OH) are present on the DE surface and considered adsorption sites.^24^ These groups are also lost through evaporation, making the DE surface more hydrophobic. Calcination is a thermal process that may lead to the loss of chemical groups related to MB adsorption, as suggested by different authors.^22–24,26^ Indeed, Yan and colleagues^26^ reported that calcined DE microspheres reached maximum MB adsorption capacity (when calcined at 600 °C) but became less efficient at adsorbing the dye as the calcination temperature increased. The authors linked the reduction in the adsorption capacity, among other factors, to the loss of hydroxyl groups during heating at higher temperatures.

In the present study, Figure 1 (Section 3.2) showed only slight changes in the FTIR bands of silanol and hydroxyl groups after DE calcination; therefore, the formation of cristobalite may be considered the most relevant factor in reducing the interaction between DE and MB molecules since the much more organized molecular arrangement in the crystalline phase may hinder intermolecular interactions. Pore-filling mechanisms can also affect DE-MB interaction.^26^ Accordingly, a surface area evaluation was conducted to fully elucidate the mechanisms responsible for the lower insecticidal activity of DE1.

The surface area study showed that DE2 has a smaller pore diameter and volume compared with the other samples (Table 3), which may be related to the inorganic and organic impurities in the sample (seen in the XRD and TG analyses) and is likely to have some impact on the insecticidal activity. However, the results suggest that surface area, pore volume, and diameter are less relevant factors compared to the crystallinity of the material in terms of insecticidal activity, considering that DE1 has a greater pore volume and diameter than DE2 but has little or no activity compared to all the samples.

**Table 3:** Specific Surface Area, Pore Volume, and Pore Diameter for Nonprocessed DE Samples

Nonprocessed samples	Specific surface area (m^2^/g)	Pore volume (cm^3^/g)	Pore diameter (nm)
DE1	27.15	0.113	8.47
DE2	20.53	0.054	5.93
DE3	22.25	0.104	9.44
DE4	20.15	0.110	12.07

## 5 Conclusion

In conclusion, DE samples from different sources (DE1, DE2, DE3, and DE4) showed significant differences in the insecticidal activity against *A. diaperinus* larvae and adults. DE4, a sample probably calcined under mild conditions, showed the best insecticidal activity, whereas DE1 showed virtually no activity. The characterization tests indicated that the DE1 sample had higher crystallinity, larger particle size, and lower adsorption and absorption capacity for methylene blue (AM). Our findings suggest that a more amorphous pattern with a smaller particle size of DE favored the insecticidal activity as well as a mild calcination process that can eliminate organic impurities without promoting crystallization. This study enables the determination of the relative contribution of each studied DE property in determining insecticidal efficacy. This understanding enables a more rational choice of raw material and helps to select the best conditions for DE processing (purification step), contributing to the development of an optimized DE-based product with low potential for insecticidal resistance, low cost, and wide availability.

## Supplementary Material



## Notes

Rietveld plot of the sample DE1 (Figure S1); Rietveld plot of the sample DE2 (Figure S2); Rietveld plot of the sample DE3 (Figure S3); Rietveld plot of the sample DE4 (Figure S4); Rietveld plot of the sample DE2-C (Figure S5); Rietveld plot of the sample DE3-C (Figure S6); and Rietveld plot of the sample DE4-C (Figure S7) (PDF)
