# Supplementary material for: Influence of the Physicochemical and Microstructural Properties on the Insecticidal Efficacy of Diatomaceous Earth on the Poultry Pest Alphitobius diaperinus
Source: ACS Omega. 2025 Oct 28;10(44):52670–80. doi: 10.1021/acsomega.5c06320 (PMC12612955; doi:10.1021/acsomega.5c06320)

Influence of the physicochemical and microstructural properties on the insecticide efficacy of diatomaceous earth on the poultry pest *Alphitobius diaperinus*

Rayssa Barbary Pedroza Moura[1], Laís Carneiro Naziasene Lima[1], Ana Lúcia Coutinho Teixeira[2], Caio Marcio de Oliveira Monteiro[2], Fabio Furlan Ferreira[3], Juliana Pereira da Silva[3], Ana Luiza Lima[4], Marcilio Cunha Filho[4], Stephânia Fleury Taveira[1], Ricardo Neves Marreto[1,*]

[1]Laboratory of Nanosystems and Drug Delivery Devices (NanoSYS), School of Pharmacy, Universidade Federal de Goiás (UFG), Goiânia, GO, Brazil

[2]Laboratory of Biology, Ecology and Tick Control, Veterinary and Zootechnics School, Universidade Federal de Goiás (UFG), Goiânia, GO, Brazil

[3]Centro de Ciências Naturais e Humanas (CCNH), Universidade Federal do ABC (UFABC), Santo André, SP, Brazil.

[4]Laboratory of Food, Drug, and Cosmetics (LTMAC), School of Health Sciences, University of Brasilia, Brasilia, DF, Brazil.

*Correspondence: Prof. Ricardo Neves Marreto. Universidade Federal de Goiás (UFG), Rua 240, Setor Leste Universitário, 74605-170. E-mail: ricardomarreto@ufg.br. Tel/Fax: +55 62 3209-6037.

Supporting information, **S1**. Rietveld plot of the sample DE1. The blue crosses indicate the observed data, while the red line indicates the calculated pattern. The gray line represents the difference between the observed and calculated patterns. The vertical bars at the bottom represent the Bragg peaks (blue: cristobalite; orange: quartz, and green: thenardite). The weight fractions are indicated within the graph.

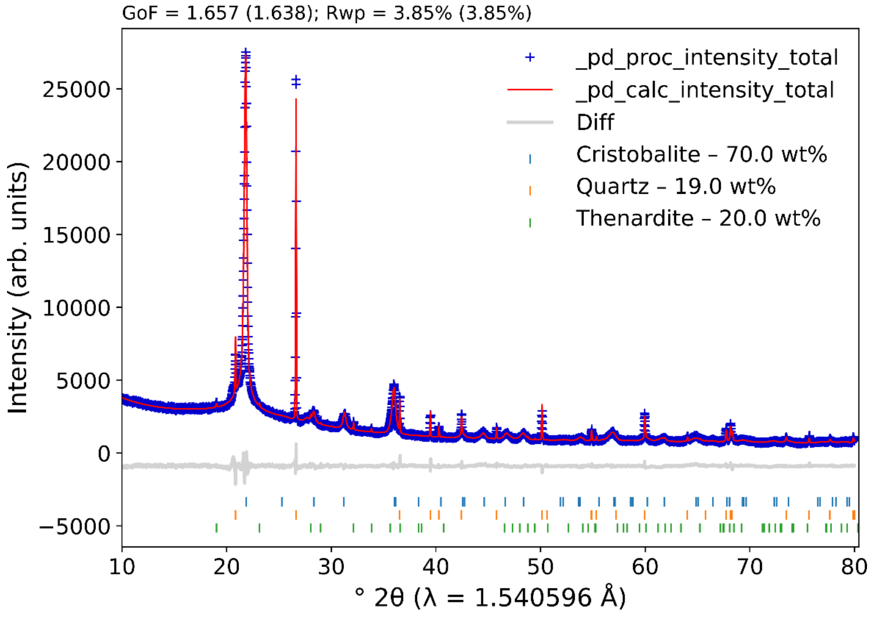

Figure S1 – Rietveld plot of the sample DE2. The blue crosses indicate the observed data, while the red line indicates the calculated pattern. The gray line represents the difference between the observed and calculated patterns. The vertical bars at the bottom represent the Bragg peaks (blue: cristobalite; orange: quartz, and green: kaolinite). The weight fractions are indicated within the graph.

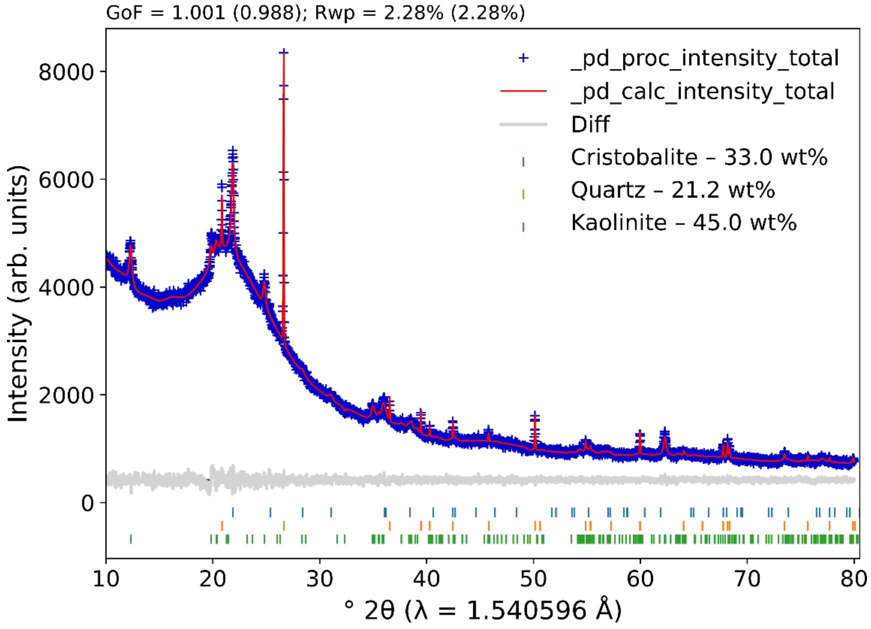

Figure S2 – Rietveld plot of the sample DE3. The blue crosses indicate the observed data, while the red line indicates the calculated pattern. The gray line represents the difference between the observed and

calculated patterns. The vertical bars at the bottom represent the Bragg peaks (blue: cristobalite; orange: quartz, and green: kaolinite). The weight fractions are indicated within the graph.

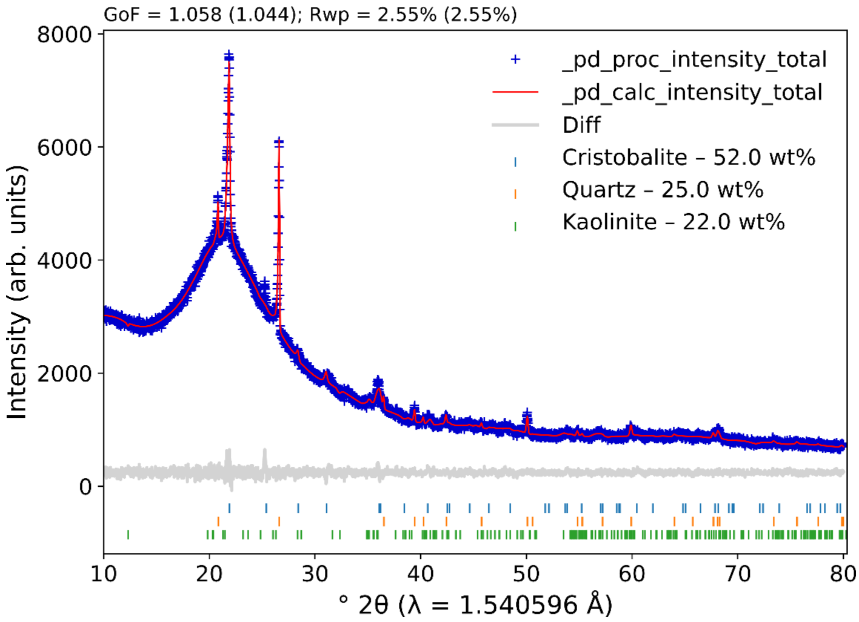

Figure S3 – Rietveld plot of the sample DE4. The blue crosses indicate the observed data, while the red line indicates the calculated pattern. The gray line represents the difference between the observed and calculated patterns. The vertical bars at the bottom represent the Bragg peaks (blue: cristobalite; orange: quartz, and green: kaolinite). The weight fractions are indicated within the graph.

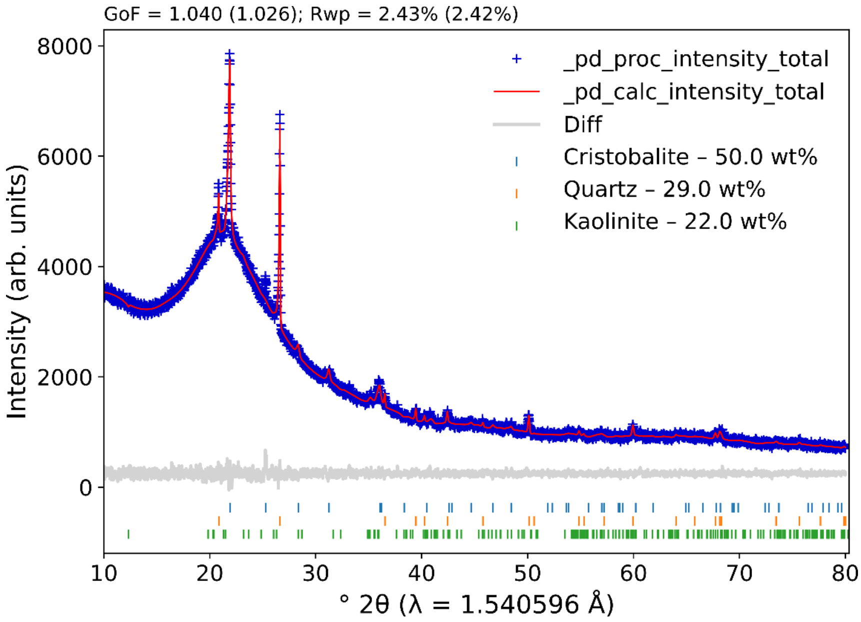

Figure S4 – Rietveld plot of the sample DE2-C. The blue crosses indicate the observed data, while the red line indicates the calculated pattern. The gray line represents the difference between the observed and

calculated patterns. The vertical bars at the bottom represent the Bragg peaks (blue: cristobalite; orange: quartz, and green: mullite). The weight fractions are indicated within the graph.

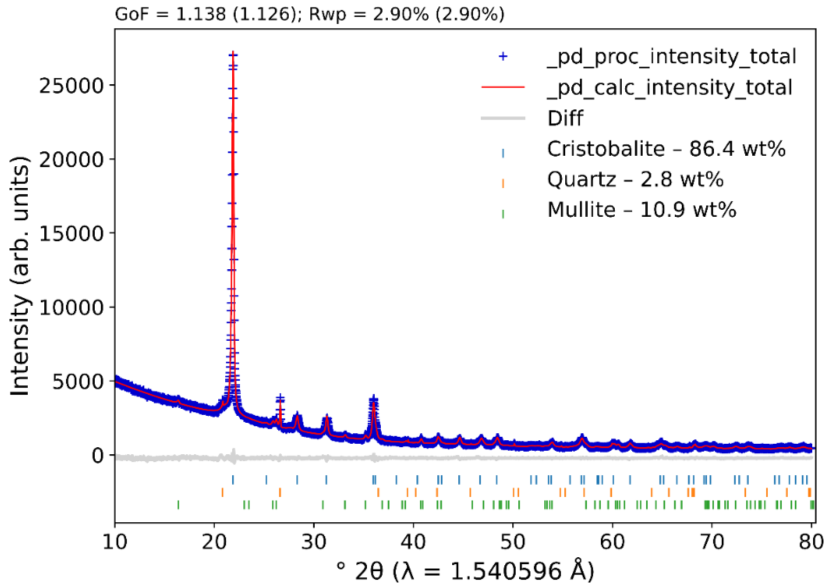

Figure S6 – Rietveld plot of the sample DE3-C. The blue crosses indicate the observed data, while the red line indicates the calculated pattern. The gray line represents the difference between the observed and calculated patterns. The vertical bars at the bottom represent the Bragg peaks (blue: cristobalite; orange: quartz, and green: mullite). The weight fractions are indicated within the graph.

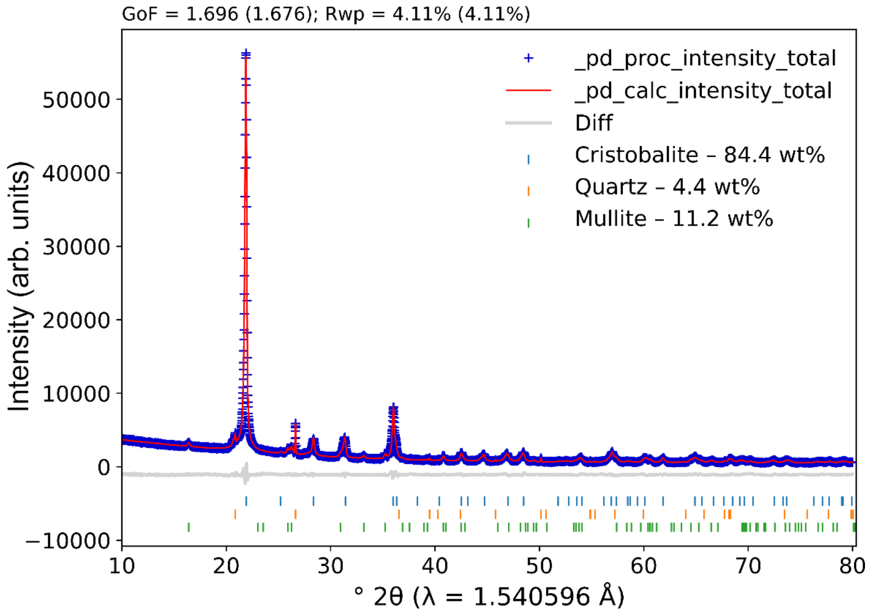

Figure S5 – Rietveld plot of the sample DE4-C. The blue crosses indicate the observed data, while the red line indicates the calculated pattern. The gray line represents the difference between the observed and calculated patterns. The vertical bars at the bottom represent the Bragg peaks (blue: cristobalite; orange: quartz, and green: mullite). The weight fractions are indicated within the graph.

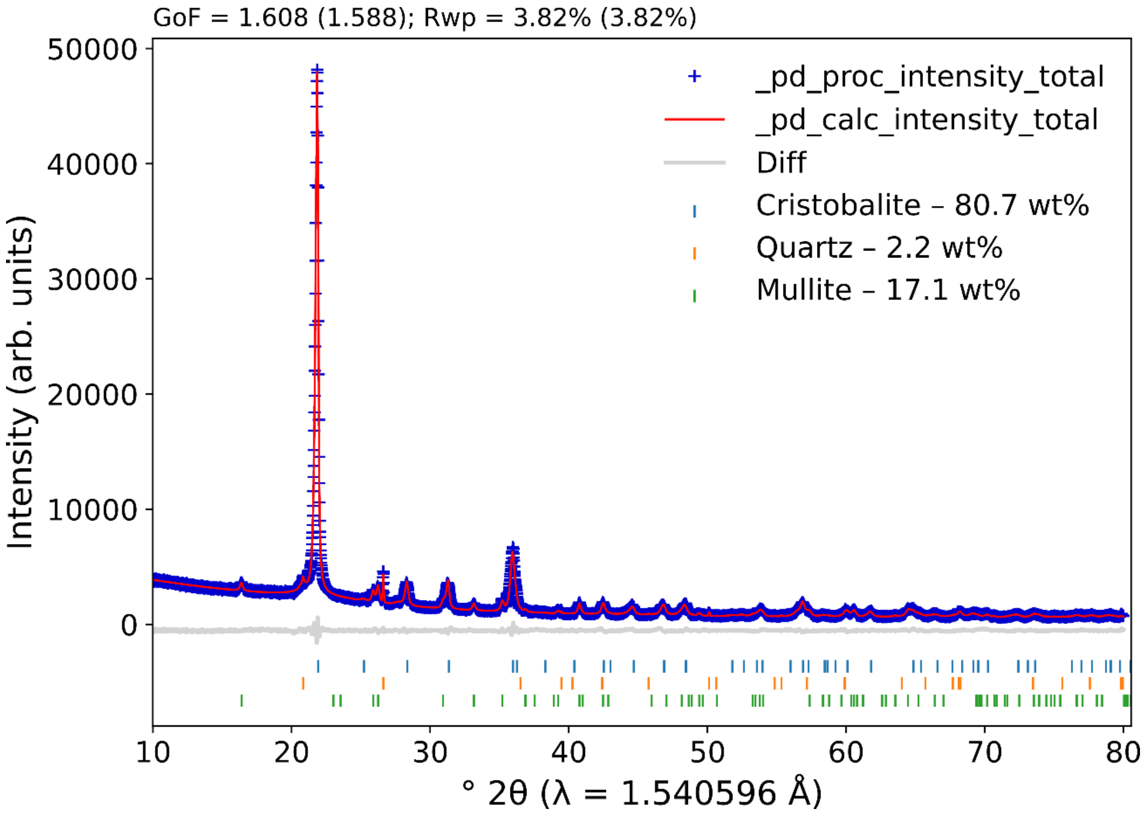

Supplement: Supplementary file 1 [file ao5c06320_si_001.pdf]
